# Psychiatric factors predict type 2 diabetes mellitus in US Veterans

**DOI:** 10.1038/s41537-025-00616-y

**Published:** 2025-04-17

**Authors:** Lora Lee Pless, Chantele Mitchell-Miland, Yeon-Jung Seo, Charles B. Bennett, Zachary Freyberg, Gretchen L. Haas

**Affiliations:** 1 VISN 4 Mental Illness Research Education and Clinical Center (MIRECC), VA Pittsburgh Healthcare System, Pittsburgh, PA USA; 2 Department of Medicine, University of Pittsburgh, Pittsburgh, PA USA; 3 Department of Statistics, University of Pittsburgh, Pittsburgh, PA USA; 4 Department of Psychiatry, University of Pittsburgh, Pittsburgh, PA USA; 5 Department of Cell Biology, University of Pittsburgh, Pittsburgh, PA USA; 6 Department of Psychology, University of Pittsburgh, Pittsburgh, PA USA

**Keywords:** Schizophrenia, Psychosis

## Abstract

Co-occurrence of type 2 diabetes mellitus (T2D) and serious mental illnesses (SMI) is prevalent yet underappreciated, and significantly contributes to increased morbidity and reduced lifespan. There is, therefore, a need to identify T2D risk factors to inform preventative approaches to the care of SMI-diagnosed patients. Our objective was to use predictive modeling methods to capture risk factors for T2D in a sample of 618,203 Veterans using data obtained from hospital electronic health records (EHR). This case-control study assessed VISN4 Veterans with and without T2D diagnoses and SMI diagnoses (schizophrenia, SZ; schizoaffective, SZA; bipolar disorder, BD; major depression, MDD; 2009-2019). Demographic variables and medications were obtained from the EHR. Following rigorous data quality control, 543,979 Veterans qualified for analysis (Age_mean[SD]_ = 65.9[17.6]years; body mass index(BMI)_mean[SD]_ = 28.6[6.0]kg/m^2^; *N*_T2D_ = 157,457[29%]; and *N*_male_ = 506,257[93.1%]). Veterans with co-occurring SMI + T2D included *N*_SZ_ = 2,087(36.5%), *N*_SZA_ = 1,345(36.3%), *N*_BD_ = 10,540(29.2%), and *N*_MDD_ = 20,510(30%) compared to 112,973(28.6%) non-SMI controls (NSC) with T2D. Factors that predicted T2D (R^2^ = 34%) included age, sex, BMI, race/ethnicity, psychiatric diagnoses, and commonly prescribed psychiatric medications. Significant interactions were found between age (centered) and BMI on the odds of T2D (*P* < 0.001), as well as interaction between sex and BMI (*P* < 0.001), after adjusting for confounders. Veterans with SMI (SZ, MDD, SZA, and BD) had a higher likelihood of experiencing T2D, compared to the NSCs (OR_SZ_ = 1.30, 95% CI = 1.21–1.40; OR_MDD_ = 1.07, 95% CI = 1.05–1.10; OR_SZA_ = 1.26, 95% CI = 1.16–1.38; OR_BD_ = 1.05, 95% CI = 1.01–1.08). Finally, Veterans exposed to both selective serotonin reuptake inhibitor (SSRI) antidepressants and mood stabilizers had a 2.11 times increase in the odds of having T2D (95% CI = 2.06–2.16; *P* < 0.001) compared to Veterans not taking either medication. Four major psychiatric disorders (SZ, SZA, MDD, and BD) and several classes of medications used to treat them increased T2D risk. Our findings suggest that the measures assayed offer a potentially useful signal, that along with clinical, anthropometric, and biochemical measures can be used to ascertain metabolic risk. If confirmed with an independent sample, these findings could also inform medication choices made by prescribers.

## Introduction

There has been a dramatic increase in type 2 diabetes (T2D) in the United States and worldwide. In 2019, the CDC estimated more than 1.4 million new cases of diabetes diagnosed in the United States^1^. Increasing evidence implicates serious mental illnesses (SMI; see Methods for definition) as significant risk factors for T2D^2–6^. Indeed, T2D increases significantly with SMI duration^7–10^, leading to major morbidity and mortality with a reduction of lifespan by 10–20 years^11,12^. Population-based studies have identified multiple risk factors for T2D, and clinical case-based research similarly suggests the involvement of multiple causal pathways in the emergence of T2D^13^. It is known that antipsychotic drugs cause metabolic disturbances^14–19^. Moreover, drug-naïve individuals diagnosed with schizophrenia (SZ) are at a 2- to 4-fold elevated risk for T2D^20–22^. Indeed, risk factors intrinsic to the biology of SZ may contribute to the development of T2D, which could accompany the risk for psychosis^16^. For example, first-degree, nonpsychotic relatives of SZ patients have an increased risk for impaired glucose tolerance^23,24^.

The identification of modifiable risk factors is an important aim to advance clinical efforts to mitigate the rise in T2D incidence in the United States. For individuals with SMI, there are several well-established modifiable risk factors that also point to etiologic mechanisms, including exposure to antipsychotic (APM) and antidepressant (ADP) medications and some mood stabilizers (e.g., lamotrigine, lithium, and carbamazepine)^25^. Epidemiologic research on risk factors for complex diseases such as T2D can benefit from predictive modeling methods that aim to capture multifactorial constellations of risk and point to possible causal mechanisms or links along putative causal pathways.

Identifying individuals with SMI at higher risk of developing comorbid T2D could guide medication choices to more effectively minimize T2D risk. Over time, efforts to appropriately match treatments to SMI patients at elevated metabolic risk could, therefore, improve health outcomes for this population. With the longer-term goal of contributing to the development of risk prediction tools for clinical application, we generated a parsimonious prediction model for T2D in this study, using well-established risk factors^26^ and classes of APM, ADP, and mood stabilizer medications commonly prescribed for patients with SMI conditions. Though prediction models for T2D have been previously proposed^27^, none have been generated using a sample size of the magnitude (>610,000 individuals) or of complex ancestral backgrounds utilized for this study. Moreover, this study is noteworthy for its focus on a Veteran population due to the elevated rate of T2D among Veterans^28,29^. Indeed, almost 1 in 4 Veterans carry a T2D diagnosis, which is more than double the T2D prevalence in the general population^28,29^.

## Methods

### Study design and database creation

The cohort included 618,203 Veterans from the VISN4 region during the time span Oct 1, 2009 to Sept 30, 2020. Once the cohort was established, medical data during FY2000-2020 for those individuals was collected. This study was approved by the institutional review board at the VA Healthcare System in Pittsburgh, PA. See the Supplemental Document for full methodological details.

### Phenotype definitions

Veterans were classified into phenotype definitions using criteria based on ICD codes and time requirements (See Supplemental Table 1 for ICD codes and requirements). We defined SMI to include the following diagnoses: SZ, schizoaffective disorder (SZA), bipolar disorder (BD), and major depressive disorder (MDD), based on similar definitions in previous studies^7,30,31^. Pharmacy records from the nationwide VA network were used to identify medication usage. Clinical records for each Veteran were used to gather information on height, weight, marital status, and ZIP code.

### Statistical analysis

#### Quality control

A total of 618,203 records were available for this study. Because these data were obtained from the electronic health records (EHR), we anticipated data entry and other types of errors. To have a complete dataset with the most accurate information, we performed rigorous quality control measures that resulted in the removal of some individuals from the cohort due to inaccurate documentation in the EHR for age, BMI, place of residence (urban/rural), sex, the reported year of death, and mismatched diagnoses for diabetes.

##### Final dataset

**Fig. 1:**
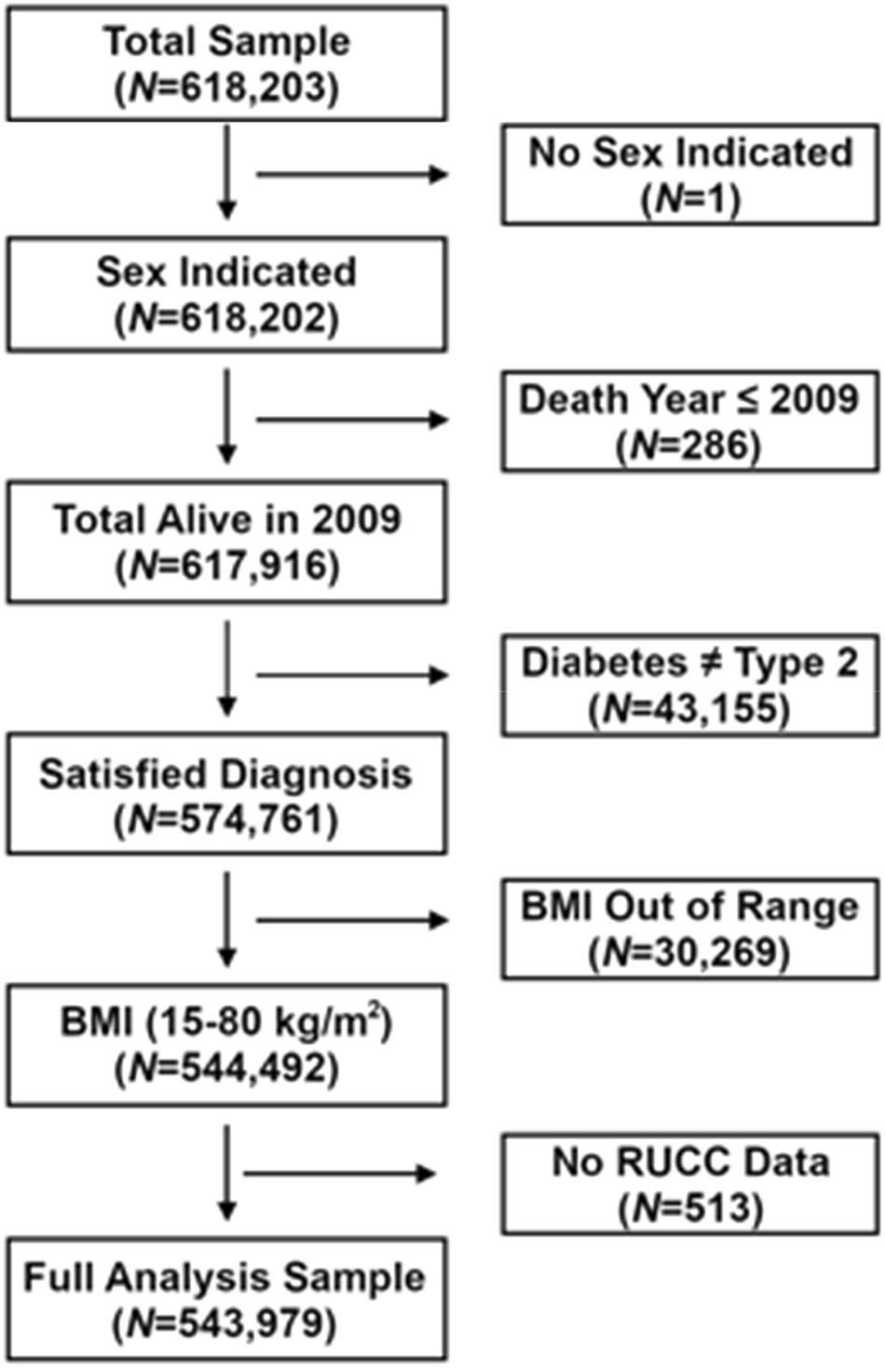
Quality control workflow diagram.

Following the removal of individuals previously described, we had a final dataset containing 543,979 Veterans (Fig. 1), which included 157,548 T2D cases, 62,688 pre-T2D, and 323,743 subjects unaffected with diabetes. See Table 1 for the demographic breakdown of the cohort. T2D prevalence was computed for the cohort. We also computed and compared the prevalence of T2D for several subgroups by sex, race/ethnicity, and psychiatric diagnosis. The numerator was the count of individuals who had a diabetes diagnosis during a given year. The denominator was the total number of Veterans who had a visit at a VISN4 healthcare facility for the year indicated (Supplemental Table 2).

**Table 1:** Cohort characteristics.

Cohort characteristics (*N*)	543,979
Demographics (% total)	
Sex (male)	506,596 (93.1%)
BMI (EHR Last Report; kg/m^2^)	28.6 [SD = 6.0]
Age (2019; years)	65.9 [SD = 17.6]
Weight (median; pounds)	199.8 [SD = 42.5]
Ancestry	
African American (AA)	68,494 (12.6%)
American Indian (AI)	1692 (0.3%)
Asian (AS)	1971 (0.4%)
European American (EA)	416,528 (76.6%)
Hispanic/Latinx (HS)	10,961 (2.0%)
Native Hawaiian/Pacific Islander (HiPac)	2531 (0.5%)
Other	41,802 (7.7%)
Marital status	
Divorced	100,233 (18.4%)
Married	296,522 (54.5%)
Never	76,291 (14.0%)
Separated	17,731 (3.3%)
Unknown	5814 (1.1%)
Widowed	47,388 (8.7%)
RUCC (Rural-urban continuum code)^#^	
Urban (RUCC 1:3)	454,837 (83.6%)
Rural (RUCC 4:9)	89,142 (16.4%)
Psychiatric phenotype (% total)	
Psychiatric diagnoses	
Bipolar disorder (BD)	36,150 (6.65%)
Major depressive disorder (MDD)	68,409 (12.58%)
Schizoaffective disorder (SZA)	3711 (0.68%)
Schizophrenia (SZ)	5728 (1.05%)
Antipsychotic medications (APM)	454,993
Generation 1 (*N* = 14 medications assessed)	31,917 (5.9%)
Generation 2 (*N* = 13 medications assessed)	63,128 (11.6%)
Clozapine	427 (0.08%)
Mood stabilizers (*N* = 8 medications assessed)	146,684 (27.0%)
Lithium	7515 (1.4%)
Antidepressants (ADP; *N* = 16 medications assessed)	212,837 (39.1%)
ADP Class^**^	
Atypical (*N* = 2 medications assessed)	67,968 (12.5%)
Monoamine oxidase inhibitor (MAO-I) (*N* = 1 medication assessed)	71 (0.01%)
Serotonin and norepinephrine reuptake inhibitor (SNRI) (*N* = 3 medications assessed)	63,377 (11.7%)
Selective serotonin reuptake inhibitor (SSRI) (*N* = 7 medications assessed)	177,849 (32.7%)
Tricyclic antidepressant (TCA) (*N* = 3 medications assessed)	22,202 (4.08%)
**Diabetes phenotype (% total)**	
Diabetes	
Type 2 diabetes mellitus	157,548 (29.0%)
Pre-type 2 diabetes mellitus	62,688 (11.5%)
Unaffected	323,743 (59.5%)
Anti-diabetic medications prescribed	
(% APMs prescribed)	
Prescriptions (*N* indiv ≥180)	117,296 (21.6%)
Prescriptions (1 ≤ *N* indiv <180)	8709 (1.6%)
Never prescribed (*N* indiv)	417,974 (76.8%)

^#^determined as the most frequently lived address.

^**^Fractions do not add to 100 due to rounding.

##### Determination of T2D prevalence

We first determined whether the prevalence of T2D was overrepresented among individuals diagnosed with psychiatric disorders by estimating the odds ratio (OR) of T2D diagnosis for individuals diagnosed with an SMI compared with the non-SMI control (NSC) group. We assessed the association groupwise by comparing SMI with NSC groups and then each SMI subgroup separately with NSCs. The ORs and the confidence intervals were calculated for each psychiatric diagnosis (SZ, SZA, BD, MDD, and SMI) using Fisher’s exact test^32^. To account for multiple comparisons, a Bonferroni correction was applied to determine significance (α = 0.05/6 tests).

##### T2D prediction model

Our goal was to generate a parsimonious model to predict T2D. One approach to model selection is by regularization of generalized linear models. Because some of the potential predictors were correlated, we chose elastic net regression (EN)^33,34^ as our model selection procedure. EN penalizes for model complexity by using the least absolute shrinkage and selection operator (LASSO) and ridge penalties (L1 and L2, respectively) on the regression coefficients. These penalty terms tend to reduce model complexity by setting some coefficients to zero and shrinking others, as well as finding robust estimates for coefficients of correlated variables, which tend to be included in or excluded from the model together (Supplemental Fig. 1). The association between phenotype variables obtained from Veteran EHR and T2D was determined using logistic regression of T2D on predictors selected by EN. The level of statistical significance was set at 0.05 (*P* < 0.05).

##### Variable selection

We considered EHR variables to build the T2D prediction model. Variables were recoded to assign comparison groups. Next, to test if there were informative interaction variables for predicting T2D, we fitted all pairwise possibilities using the 18 EN-selected. All main effect variables were forced into the model. Interactions were retained in the model if they were significant. In addition to these variables identified by EN, literature reports an increased risk of T2D for African American (AA) women^35–37^. The interactions between sex and race/ethnicity were, therefore, forced into the final model. All analyses were performed using R statistical software (version 4.3.1)^38^.

##### Inclusion and ethics statement

All authors of this study have fulfilled the criteria for authorship required by Nature Portfolio journals. Their participation was essential for the design and implementation of the study.

## Results

### Odds ratios of T2D

The unadjusted ORs of T2D were estimated for the following variables: sex, race/ethnicity, and psychiatric diagnoses. **Sex.** Males were associated with a 2.6-fold increased odds of T2D compared to females (OR_Males_ = 2.63, 95% CI = 2.56–2.71, *P* = 0). **Race/ethnicity**. Compared to the EA group (baseline), the AA group was associated with 1.3-fold increased odds of T2D (OR_AA_ = 1.32, 95% CI = 1.29–1.34, *P* = 7.3 × 10^−194^). HS, AS, and other race/ethnicity groups were associated with decreased odds of T2D (OR_HS_ = 0.93, 95% CI = 0.89–0.97, *P* = 1.5 × 10^−03^; OR_AS_ = 0.45, 95% CI = 0.40–0.51, *P* = 1.39 × 10^−45^; OR_Other_ = 0.85, 95% CI = 0.83–0.87, *P* = 2.14 × 10^−43^). The remaining race/ethnicity groups (AI and HiPac) were not associated with odds of T2D, when considering a Bonferroni corrected *P* < 0.008 (α = 0.05/6 tests): OR_AI_ = 0.89, 95% CI = 0.80–1.0, *P* = 0.04; OR_HiPac_ = 1.07, 95% CI = 0.98–1.17, *P* = 0.11. **Psychiatric diagnostic groups***.* Compared to the NSC group (baseline), SMI was associated with ~1.1-fold increased odds of T2D (OR_SMI_ = 1.07, 95% CI = 1.06–1.09, *P* = 3.4 × 10^−20^). Furthermore, we found SZ, SZA, and MDD were associated with 1.6-, 1.5-, and 1.1-fold increased odds of T2D, respectively (OR_SZ_ = 1.61, 95% CI = 1.52-1.71, *P* = 4.3 × 10^−58^; OR_SZA_ = 1.47, 95% CI = 1.37–1.57, *P* = 4.02 × 10^−26^; OR_MDD_ = 1.06, 95% CI = 1.04−1.08, *P* = 5.1 × 10^−10^). We did not identify this association with BD (OR_BD_ = 1.0, 95% CI = 0.97–1.02, *P* = 0.59). The prevalence of T2D among these subgroups are depicted in Supplemental Table 2. **Psychiatric medications.** Of the medications that subjects were exposed to, the most common were gabapentin (*N* = 136,951; mood stabilizer), and antidepressants trazodone and sertraline (*N* = 109,422 and 99,367, respectively). The most common second-generation APMs were quetiapine, risperidone, and aripiprazole (*N* = 46,046, 28,234, and 25,289, respectively; Supplemental Table 1).

### T2D prevalence

**Fig. 2:**
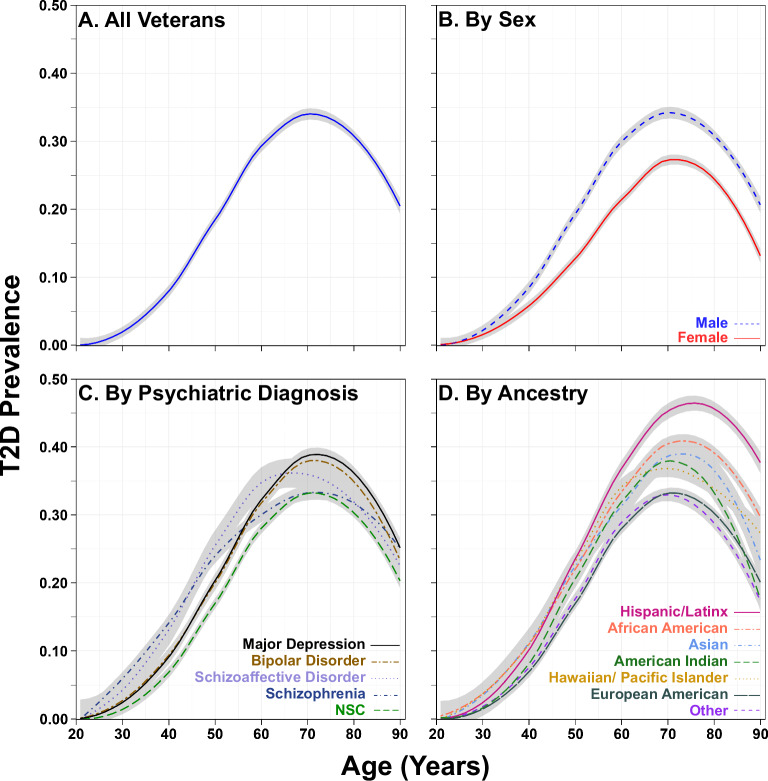
Prevalence of type 2 diabetes mellitus in VISN4 veterans: year 2019. **A** Prevalence of T2D in the full VISN4 cohort, **B** Prevalence of T2D per males and females, **C** Prevalence of T2D per psychiatric diagnostic group, **D** Prevalence of T2D among Veterans per ancestry group. T2D prevalence was determined for Veterans during the year 2019. The total number of Veterans with a confirmed T2D diagnosis was the numerator, and the total number of Veterans visiting a VISN4 facility for medical care was the denominator. A Loess smoothing line was fit to display T2D prevalence, and the 95% confidence interval was displayed as gray shading. T2D, type 2 diabetes mellitus; NSC, non-SMI controls.

The prevalence of T2D for the cohort in 2019 was 25.2%. The 2019 prevalence was also computed for the cohort by age, sex, psychiatric diagnosis, and race/ethnicity (Fig. 2). All psychiatric groups displayed a higher average prevalence of T2D compared to the NSC group across all age groups (Fig. 2A). Individuals diagnosed with depression or BD showed similar patterns of T2D prevalence and after the mid-sixth decade, had the highest prevalence of T2D. We observed differential patterns of T2D prevalence among psychiatric groups, most notably for SZ and SZA, which showed an increased prevalence of T2D earlier than other groups. These profiles were similar until the individuals’ mid- to late-40s, after which the prevalence of T2D increased for SZA over SZ. The prevalence of T2D in the SZ group steadily increased until the age of 60, where it reached a plateau before decreasing in the 8th decade. For the SZA group, the prevalence of T2D continued to increase, albeit at a lower slope than the SZ group (Fig. 2C). The prevalence of T2D was further stratified by race/ethnicity. At the apex (age 75), we found the highest prevalence of T2D for individuals identified as HIS, followed by AA, AS, and AI groups (Fig. 2D).

### Prediction logistic regression model

Supplemental Fig. 1 shows the EN with tenfold cross-validation that was used for regularization and to select variables among those that were significant predictors of T2D. More detailed information for EN procedure and tuning parameters are summarized in the Supplemental Document. Parameter estimates from fitting the logistic regression model are described in Table 2.

**Table 2:** Type 2 diabetes mellitus prediction model.

		Variable	*N*	Estimate	SE	OR (95% CI)	Partial Pseudo *R*^2^	*P*
**Main effect variables**	**Demographic**	Age (std-mean)	543,979	0.058	0.000	1.060 (1.059—1.061)	1.00 × 10^−01^	0
		Age (std-mean)^2^	543,979	−0.002	0.000	0.998 (0.998—0.998)	4.25 × 10^−01^	0
		Race/ethnicity (AA)	68,494	0.609	0.012	1.838 (1.796—1.881)	8.71 × 10^−02^	0
		Race/ethnicity (AI)	1692	0.119	0.068	1.127 (0.987—1.287)	1.01 × 10^−05^	7.83 × 10^−02^
		Race/ethnicity (AS)	1971	0.867	0.078	2.379 (2.041—2.774)	3.68 × 10^-04^	1.66 × 10^−28^
		Race/ethnicity (HiPac)	2531	0.260	0.053	1.297 (1.170—1.439)	7.85 × 10^-05^	8.68 × 10^−07^
		Race/ethnicity (HS)	10,961	0.517	0.027	1.677 (1.589—1.770)	1.15 × 10^−03^	1.91 × 10^−79^
		Race/ethnicity (Other)	41,802	−0.049	0.014	0.952 (0.927—0.978)	4.33 × 10^−05^	2.92 × 10^−04^
		BMI	543,979	0.805	0.004	2.237 (2.218—2.256)	1.27 × 10^−01^	0
		Marital status (Divorced)	100,233	−0.023	0.010	0.978 (0.959—0.997)	1.75 × 10^−05^	2.13 × 10^−02^
		Marital status (Never)	76,291	0.000	0.013	1.000 (0.975—1.026)	6.52 × 10^−10^	9.89 × 10^−01^
		Marital Status (Single)	17,731	0.020	0.021	1.020 (0.978—1.064)	2.86 × 10^−06^	3.51 × 10^−01^
		Marital Status (Unknown)	5814	−0.594	0.044	0.552 (0.506—0.602)	6.44 × 10^−04^	4.21 × 10^−41^
		Marital Status (Widow)	47,388	0.083	0.012	1.087 (1.061—1.113)	1.52 × 10^−04^	9.69 × 10^−12^
		Sex (F)	37,383	−0.546	0.024	0.579 (0.553—0.607)	1.85 × 10^−03^	7.10 × 10^−115^
		Urban residence	454,837	−0.148	0.009	0.862 (0.847—0.878)	8.13 × 10^−04^	5.63 × 10^−56^
	**Psychiatric Disorder**	Bipolar disorder	36,150	0.045	0.018	1.046 (1.011—1.083)	2.16 × 10^−05^	1.04 × 10^−02^
		Major depression	68,409	0.069	0.013	1.072 (1.045—1.099)	9.27 × 10^−05^	1.08 × 10^−07^
		Schizoaffective disorder	3711	0.234	0.044	1.264 (1.159—1.379)	9.12 × 10^−05^	1.33 × 10^−07^
		Schizophrenia	5728	0.261	0.036	1.298 (1.210—1.393)	1.74 × 10^−04^	3.03 × 10^−13^
	**Psychiatric medication**	ADP (Atypical)	67,968	0.048	0.013	1.049 (1.023—1.076)	4.55 × 10^−05^	2.00 × 10^−04^
		Lithium	7515	−0.138	0.035	0.871 (0.813—0.933)	5.14 × 10^−05^	8.20 × 10^−05^
		ADP (MAO-I)	71	−0.193	0.290	0.824 (0.467—1.456)	1.47 × 10^−06^	5.05 × 10^−01^
		ADP (Mood stabilizer)	146,684	0.786	0.012	2.194 (2.145—2.244)	1.51 × 10^−02^	0
		ADP (SNRI)	63,377	0.124	0.013	1.132 (1.103—1.161)	2.98 × 10^−04^	1.53 × 10^−21^
		ADP (SSRI)	177,849	0.309	0.011	1.362 (1.334—1.392)	2.60 × 10^−03^	3.62 × 10^−176^
		ADP (TCA)	22,202	0.234	0.019	1.264 (1.219—1.312)	5.14 × 10^−04^	5.33 × 10^−36^
		Clozapine	427	0.530	0.127	1.699 (1.325—2.177)	5.76 × 10^−05^	2.87 × 10^−05^
		APM (Generation 1)	31,917	0.250	0.015	1.284 (1.246—1.323)	8.70 × 10^−04^	8.22 × 10^−60^
		APM (Generation 2)	63,128	−0.041	0.014	0.960 (0.934—0.987)	2.70 × 10^−05^	4.21 × 10^−03^
**Interaction variables**		ADP (mood stabilizer) × ADP (SSRI)	89,482	−0.348	0.017	0.706 (0.683—0.730)	1.36 × 10^−03^	6.07 × 10^−92^
		BMI × Age	543,979	−0.004	0.000	0.996 (0.996—0.997)	5.23 × 10^−04^	2.49 × 10^−36^
		BMI × Sex (F)	37,383	−0.095	0.016	0.910 (0.881—0.939)	1.08 × 10^−04^	7.25 × 10^−09^
		Sex (F) × Race/ethnicity (AA)	8728	0.113	0.041	1.120 (1.033—1.214)	2.47 × 10^−05^	6.04 × 10^−03^
		Sex (F) × Race/ethnicity (AI)	209	−0.034	0.261	0.966 (0.580—1.610)	5.70 × 10^−08^	8.95 × 10^−01^
		Sex (F) × Race/ethnicity (AS)	315	0.540	0.230	1.715 (1.092—2.694)	1.70 × 10^−05^	1.92 × 10^−02^
		Sex (F) × Race/ethnicity (HiPac)	239	0.573	0.210	1.773 (1.175—2.674)	2.34 × 10^−05^	6.32 × 10^−03^
		Sex (F) × Race/ethnicity (HS)	1316	0.203	0.105	1.226 (0.998—1.505)	1.22 × 10^−05^	5.19 × 10^−02^
		Sex (F) × Race/ethnicity (Other)	3064	0.176	0.069	1.193 (1.041–1.366)	2.09 × 10^−05^	1.09 × 10^−02^

A logistic regression model was used to predict T2D using variables obtained from electronic health records of Veterans in the VISN4 region. The comparison groups used in this model were as follows: for Race/ethnicity, EA, for marital status, married, for psychiatric diagnosis, NSC, for residence, rural, and for sex, male. Race/ethnicity: AA, African American, AI, American Indian, AS, Asian, EA, European American, HS, Hispanic, HiPac, Hawaiian/Pacific Islander. N, count of individuals, se, standard error; P, *p* value; OR (95% CI), odds ratio (95% confidence interval). Note, *P* = 0 indicates the exponent is less than 1 × 10^−308^.

In our study, the odds of T2D were estimated to be lower in urban areas than in rural areas (OR_Urban_ = 0.86, 95% CI = 0.85–0.88, *P* < 0.001). In addition, compared to married veterans, widowed status was associated with higher odds of T2D (OR_Widowed_ = 1.09, 95% CI = 1.06.0–1.11, *P* < 0.001), whereas being divorced was associated with slightly lower odds of T2D (OR_Divorced_ = 0.98, 95% CI = 0.96.0–1.00, *P* = 0.021).

Significantly, our model both validated and expanded upon the links between SMIs and metabolic disease. We found that Veterans with psychiatric disorders (SZ, SZA, MDD, and BD) had higher odds of T2D, compared to the NSCs (OR_SZ_ = 1.30, 95% CI = 1.21–1.40; OR_SZA_ = 1.26, 95% CI = 1.16–1.38; OR_MDD_ = 1.07, 95% CI = 1.05–1.10; OR_BD_ = 1.05, 95% CI = 1.01–1.08). We also observed that among all, those who were treated with SSRIs and mood stabilizing medications (excluding lithium) had 2.11-fold higher odds of T2D (95% CI = 2.06–2.16; *P* < 0.001) compared to those not taking either medication. The clinical and demographic characteristics of Veterans who took both SSRIs and mood stabilizers are shown in Supplemental Table 3. Each of the interactions between sex and race/ethnicity (AA, AS, HiPac, and Other) were statistically significant: for example, compared to the male EA group, the male AA group was associated with 1.84-fold increased odds of T2D, while controlling for other covariates (95% CI = 1.80–1.88; *P* < 0.001). On the other hand, the female AA group was associated with 2.06-fold increased odds of T2D (95% CI = 1.90–2.23; *P* < 0.001) compared to the female EA group. We found the interactions between BMI and age and between BMI and sex had significant effects on the odds of T2D (both *P* < 0.001). For example, for male veterans with a mean age of 65.9 years, the odds of T2D increased by about 124% for each BMI unit increase (OR_BMI,Male_ = 2.24, 95% CI = 2.22–2.26; *P* < 0.001), while for females, T2D odds increased by 100% for each BMI unit increase (OR_BMI,Female_ = 2.03, 95% CI = 1.97.0-2.10; *P* < 0.001). In contrast, the use of lithium or APM (generation 2) medications were each associated with a reduction in the odds of T2D (OR_Lithium_ = 0.87, 95% CI = 0.81–0.93, *P* < 0.001; OR_ADP-Generation2_ = 0.96, 95% CI = 0.93-0.99, *P* = 0.004), while the other medications were associated with increased odds of T2D—with the exception of MAO-I, which was not associated with T2D risk.

## Discussion

It has long been observed that individuals diagnosed with SMI have an increased risk of developing T2D^2,3^. The current study represents one of the largest study samples to date. In harnessing the power of this sample size, we not only confirmed previously identified risk factors for T2D, but also extended prior findings to evaluate the contributions of different families of psychopharmacologic agents to T2D. As an additional advance, we utilized EN to interrogate all pairwise interactions for association with T2D within a large regional sample. Using EN regression, we found that BMI, age, sex, some race/ethnicity categories, and psychiatric diagnosis (SZ, SZA, BD, and MDD) contributed significantly to T2D risk in this sample. In addition to these variables, urbanicity and certain classes of psychopharmacologic medications were significant contributors to T2D risk. We also confirmed that interactions between BMI and age, BMI and sex, and interactions between sex and some of the race/ethnicity categories^35,36^ were important predictors of T2D. Surprisingly, we discovered that the interaction of mood stabilizer and SSRI use also contributed to the T2D prediction model.

**Fig. 3:**
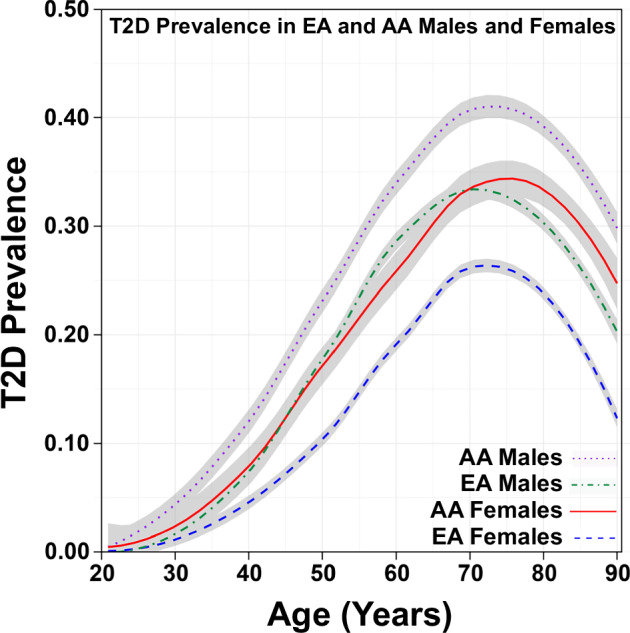
Prevalence of type 2 diabetes mellitus in VISN4 veterans for men and women of African American or European American ancestry. T2D prevalence was determined for veterans during the year 2019. The total number of veterans with a confirmed T2D diagnosis was the numerator, and the total number of veterans visiting a VISN4 facility for medical care was the denominator. A Loess smoothing line was fit to display T2D prevalence, and the 95% confidence interval was displayed as gray shading.

Our findings were consistent with other reports regarding age, race/ethnicity, and psychiatric diagnoses. Younger Veterans (aged 20 years or less) had a T2D prevalence of <1%; however, this estimate increased to nearly 40% for Veterans aged 70+ years. While the pattern of T2D prevalence by age was similar for males and females, the prevalence rates by age were higher for males. Furthermore, we found differences in T2D prevalence for self-reported race/ethnicity groups. The race/ethnicity groups displayed similar prevalence profiles by age, albeit magnitudes were substantially different for some groups. Consistent with a recent CDC study of >560,000 New York City residents, individuals identifying as HS, AA, and AS had the highest prevalence of T2D in this cohort and followed a similar T2D prevalence profile by age as the male Veterans in this study^39^. Prior studies indicated a significant interaction between sex and race/ethnicity for T2D prevalence, primarily for AA females as compared with EA males^35–37^. Thus, we also explored the effect of sex and race/ethnicity for the two most commonly reported race/ethnicity groups: EA and AA (Fig. 3). For each age group, AA males had the highest prevalence of T2D, as compared with EA males, EA females, and AA females. Furthermore, AA females and EA males had nearly overlapping prevalence rates across all ages, with the exception of a divergence from age 70 to age 90, where AA female prevalence exceeded that for EA males—perhaps because AA females live longer. Together, this is largely consistent with prior work demonstrating an increased prevalence of T2D and poorer clinical outcomes for AA females^35^, as well as for AA individuals^36^.

In this study, mood stabilizers and SSRIs were significant risk factors for T2D. Indeed, a history of exposure to both (represented by the interaction of mood stabilizing medications and SSRIs) was associated with a ~2-fold increased prevalence of T2D compared to individuals not using these medications (Table 2 and Supplemental Fig. 2). Each of these classes of medications can increase the risk for T2D via drug-induced weight gain or disturbances in molecular pathways required for glycemic control^40,41^. Whether exposure to both has a synergistic or additive effect is unclear. However, the actions of these medications are complex given their concurrent actions both in the central nervous system and systemically^40–43^. Therefore, more work is required to decipher the effects of both ADP and mood-stabilizing drugs at a mechanistic level.

Similarly, the effects of first- versus second-generation APMs are complicated as they have very different metabolic profiles, in part due to their different receptor targets. Although second-generation APMs are traditionally associated with worse metabolic profiles, in this study, it was observed that first-generation, not second-generation, APMs were associated with an increased risk of T2D. Though this finding is somewhat counterintuitive, it is possible that these results may be a function of the chronicity of illness. It is also possible that subjects who were prescribed first-generation APMs represent individuals who have lived with SMIs and have been treated for longer time periods, as compared to more recently diagnosed individuals (who were more likely to have received second-generation medications). If so, treatment with first-generation APMs may represent the longer duration of APM treatment and thus, could confer a higher cumulative metabolic burden and overall T2D risk than second-generation APMs. Furthermore, lithium-treated subjects were associated with a reduction in the odds of T2D. This is consistent with earlier studies, which demonstrated that lithium produced either no change in glycemic control and/or T2D risk, and, in some cases, reduced risk^44–46^.

Importantly, in our model, in contrast to the other psychiatric diagnoses, BD was minimally associated with T2D, after controlling for other covariates. In this sample, it was observed that mood stabilizing medications (excluding lithium) prescribed for BD weighed more heavily in association with T2D than did BD diagnosis. Further research is needed that is directed to the question of risk for T2D in BD. Moreover, caution should be used in prescribing medications that are associated with weight gain or other metabolic side effects^47^.

The etiology of T2D is complex and likely involves several underlying neurobiologic mechanisms contributing to resistance to insulin—mechanisms that (in concert with metabolic dysfunction in the periphery) may culminate in impaired release of insulin (due to defective pancreatic beta cell function). Our findings indicate that Veterans diagnosed with some SMIs carry a higher risk of developing T2D compared to NSC, consistent with prior reports^9,10,48^. However, the nature of the links between SMI and diabetes remains unknown. Recent genome-wide association studies for a variety of SMIs and diabetes suggest DNA variants are associated with each disease across the entire genome^49–52^. As for another complex human disease, the risk for diabetes is likely due to a combination of genetic and environmental risk factors, as suggested by the multifactorial polygenic threshold theory. Identification of these multiple risk factors in future research could not only explain the etiology of these co-occurring disorders, but also guide the future development of biomarkers of risk for T2D^53^.

### Limitations

The prediction model generated by this work is limited by the following: first, this sample is largely comprised of EA males. Thus, this is not faithfully representative of the general US population, nor necessarily of the population of Veterans living in areas outside of the VISN4 region. Second, this study cannot determine the causal nature of the associations between diabetes and medication use and/or psychiatric disease. Furthermore, the dating of the onset of T2D and SMI disorders was not available for this cohort; the temporal overlap of these disorders could not be adequately assessed. Third, the EHR records did not contain family history or information on lifestyle factors, such as diet, physical activity, socioeconomic status, or level of education, which are important factors for T2D. Thus, we were unable to account for these factors in our prediction model. In addition, we were unable to evaluate within-subject longitudinal risk because we did not have access to T2D age-of-onset for all cases—however, we observed that the prevalence of T2D increased from age 20 to later in life, and then dropped significantly, in part, due to increasing T2D-related mortality with age. Furthermore, the quantitation of medication exposure was limited; medication adherence could not be taken into account and exposure was coded simply in terms of presence/absence over lifetime. It is also possible that some of the subjects defined as SMI had comorbid conditions such as posttraumatic stress disorder, substance use disorder, or personality disorders. These conditions could also be associated with weight gain and T2D. Another limitation is that our use of race/ethnicity could be a proxy for other measures, such as socioeconomic status and level of deprivation; these measures were not available and thus could not be included as potential confounders in the models. Finally, the present study was unable to assess the co-occurrence of first and second-generation APMs. Future studies are needed to investigate the impact of medication exposure by considering variables like medication class, receptor binding properties, and dosing in order to better determine how medications relate to T2D risk. Finally, the role of posttraumatic stress disorder or non-psychiatric medications known to be associated with T2D should be considered in future work.

## Conclusion

Ultimately, we identified significant risk factors to predict T2D in a sample size that exceeds a half-million individuals while considering psychiatric diagnoses and medication use. Findings from this study open the door to future work that examines the role of aging and medication exposure, in risk for T2D, independent of psychiatric diagnosis. A better understanding of these risk factors will enable the development of strategies for controlling or preventing metabolic syndromes, like T2D, in vulnerable populations. Future studies that demonstrate the feasibility and utility of such prediction models may help guide healthcare decisions.

## Supplementary information

Supplemental Methods

Supplemental Table 1

Supplemental Table 2

Supplemental Table 3

Supplemental Figures 1 and 2

## Data Availability

The study participants did not provide consent for protected health data to be shared publicly; given the sensitive nature of the variables used in this study, data were not publicly available.

## Code availability

The study participants did not provide consent for protected health data to be shared publicly; given the sensitive nature of the variables used in this study, code are not publicly available.
