# Supplementary material for: Psychiatric factors predict type 2 diabetes mellitus in US Veterans
Source: Schizophrenia (Heidelb). 2025 Apr 17;11(1):63. doi: 10.1038/s41537-025-00616-y (PMC12003899; doi:10.1038/s41537-025-00616-y)
Supplement: Supplementary file 1 — Supplemental Methods [file 41537_2025_616_MOESM1_ESM.docx]

Supplemental Document.

**Methods**.

The cohort was defined as all Veterans with >1 outpatient encounter days in a VISN 4 VA facility during fiscal years (FY) 2010-2019 (calendar years: Oct 1, 2009 to Sept 30, 2020). VISN 4 VA medical facilities were located in Altoona, Butler, Philadelphia, Pittsburgh, Erie, Lebanon, Wilks-Barre, and Coatesville (Pennsylvania) and Wilmington (Delaware). An encounter was defined as an outpatient/inpatient record on a given date; multiple records recorded on the same day were counted as a single encounter day. Once the cohort was established (*N*=618,203 Veterans), medical data during FY2000-2020 for those individuals was collected. Cohort membership was defined on a year-by-year basis, and it was necessary for a given Veteran to have at least two encounters more than 30 days apart, but within 390 days to be included in the cohort for that year. Since some Veterans visit VA facilities located in different VISNs, we examined encounters from the nation-wide VA network for phenotyping in addition to those encounters in a VISN 4 facility.

**Phenotype Definitions**

Diabetes. Diabetes was identified using ICD9 and ICD10 codes. Individuals with confirmed T2D required ≥2 VA encounters with a T2D diagnosis code that were at least 30 days apart and not more than 390 days apart. We used 390 days, instead of 365, to allow for annual exams that may not happen exactly within one year (termed "time rule" in this manuscript). If the individual also had a T1D diagnosis in addition to T2D diagnoses, the individual was classified as "mixed diagnosis." If an individual had a single T2D diagnosis without a confirming second diagnosis, he or she was categorized as "unconfirmed." Individuals with confirmed T1D, mixed, or unconfirmed diagnoses were not considered T2D, were not included in the T2D group, and were excluded from the T2D control group. Pre-T2D status was defined as follows: i) hemoglobin A1C (HbA1c) levels within the range of 5.7%-6.4% at any point in the individual's EHR, ii) did not meet criteria for T1D or T2D, and iii) had no history of antidiabetic medication from the pharmacy benefits management records. These individuals were not used in prediction modeling.

Psychiatric Disorder Diagnoses. We identified individuals who were diagnosed with SMI (SZ, SZA, BD, MDD), or none of these (non-SMI controls, "NSC"). We first classified individuals into the SZ, SZA, and BD groups using the greatest number of recorded ICD diagnoses as the primary psychiatric diagnosis and the aforementioned time rule. In the case of ties, individuals were classified into one of the three diagnosis groups using the following order: SZ > SZA > BD. For the remaining individuals, we assigned MDD diagnosis using ICD9 -10 codes and the time rule. Individuals who were not classified into one of these four groups belonged to the NSC group.

Psychiatric Medications**.** The use of APMs was tabulated per individual. We identified two groups of APMs: APM-generation 1, APM-generation 2, as well as clozapine, mood stabilizers, antidepressants, and Lithium. Each Veteran was coded as a binary variable (ever/never) for each type. In addition, we defined six subgroups for medications commonly prescribed for major depression (atypical, selective serotonin reuptake inhibitors "SSRI", serotonin and norepinephrine reuptake inhibitors "SNRI", monoamine oxidase inhibitor "MAO-I", and tricyclic). See Supplemental Table 1 for listings of diagnostic codes and prescription medication group membership.

**Statistical Analysis**

**Quality Control**

Phenotyping Variables. **Ancestry.** Self-reported race and ethnicity was converted to a single variable and referred to henceforth as "ancestry". Individuals were identified as: African American (AA), American Indian (AI), Asian (AS), European American (EA), Hispanic/Latinx (HS), Native Hawaiian or Pacific Islander (HiPac), Mixed, and Other. Individuals classified as unknown ancestry did not have a reported race/ethnicity reported in their EHR. These individuals, along with Veterans reporting "mixed", were subsequently grouped together in a group termed "other." No individuals were removed due to unknown or any other category of ancestry. **Age.** The age used was that person's age on July 1, 2019, or at death, if death was prior to that date. Individuals with age>90 years were coded as 90. **Marital Status.** The marital status was self-reported and recorded as divorced, married, never married, separated, widowed, or unknown. No subjects were removed for marital status non-membership. **Body Mass Index (BMI).** The median BMI (kg/m^2^) was determined (2009-2019). We retained Veterans with 15<BMI<80 (30,619 individuals removed). **Rural-Urban Continuum Codes.** The RUCC is a scale of 1-9, where levels 1-3 are considered urban (or metro) and levels 4-9 are considered rural (or nonmetro), as determined by the United States Department of Agriculture.^1^ The RUCC were determined per Veteran, based on county of residence, and then converted to a binary variable (urban or rural). There were 513 Veterans removed due to missing values. **Sex.** Each Veteran had either male or female designated; however, one person did not specify sex in the EHR and was thus removed from analysis. **Death Year.** The year of death was recorded for deceased Veterans. We removed 286 individuals whose records contained nonsensical death years (*e.g*., Veterans who had a death year recorded, but also had a medical record entry for care after the recorded date of death). This indicated data entry or another type of error that we could not rectify. **Diabetes.** There were 43,155 individuals removed for unconfirmed T2D diagnosis; their EHR indicated 1) both T1D and T2D, or 2) did not meet the time rule requirement.

Data Coding. All variables were coded as binary, with the exception of selected indicator variables (ancestry, marital status, and psychiatric diagnosis), and continuous variables (age and BMI). Continuous variables were standardized by subtracting the mean value for each subject.

Main Effect Variable Selection.

The comparison (baseline) groups for ancestry was EA, for marital status was married, for psychiatric diagnosis was NSC, and for sex was male. These groups were chosen because they represented the largest count of individuals per variable. Individuals considered pre-T2D were excluded; the outcome was T2D (yes/no). A quadratic term for (centered) age was included in the model selection procedure which was shown to be significant. (Supplemental Figure 1). The EN with 10-fold cross validation was used to select variables among those that were significant predictors of T2D. The lambda value that gave the minimum mean cross-validated error was (0.0001, λMIN) and the lambda value that gave the most regularized model with cross-validated error within one standard error of the minimum was (0.0006, λ1SE). We chose the more conservative outcome, λ1SE, which identified all variables as statistically significant, except for three categories within the indicator variables: BP diagnosis and marital statuses: "divorced" and "never." The fraction of variance explained by this model for predicting T2D was 32.32%. The top five variables explained >96% of the model variance: BMI, age, age2, mood stabilizers, and AA ancestry (Table 2).

Data Programs and R Packages.

T2D prevalence estimates were conducted using the 'epitools' package^2^. For model selection (main effect and interactions), we performed EN using the 'glmnet' package (version 4.1-3)^3^. The variance explained by the model was determined using Nagelkerke's pseudo R^2^ for the overall model and for each variable in the model (partial pseudo R^2^) using 'fmbs' package (version 0.7.3).

WORK CITED.

(1) McGranahan, D.; HJC; Mines, F.; Jordan, M. Social and Economic Characteristics of the Population in Metro and Non- Metro Counties, 1970-80., 1986.

(2) Aragon, T. *Epitools: Epidemiology Tools. R Package Version 0.5-10.1*.

(3) Friedman, J.; Hastie, T.; Tibshirani, R. Regularization Paths for Generalized Linear Models via Coordinate Descent. *J. Stat. Softw.* **2010**, *33* (1), 1–22.
