# Supplementary material for: Psychiatric factors predict type 2 diabetes mellitus in US Veterans
Source: Schizophrenia (Heidelb). 2025 Apr 17;11(1):63. doi: 10.1038/s41537-025-00616-y (PMC12003899; doi:10.1038/s41537-025-00616-y)
Supplement: Supplementary file 5 — Supplemental Figures 1 and 2 [file 41537_2025_616_MOESM5_ESM.pdf]

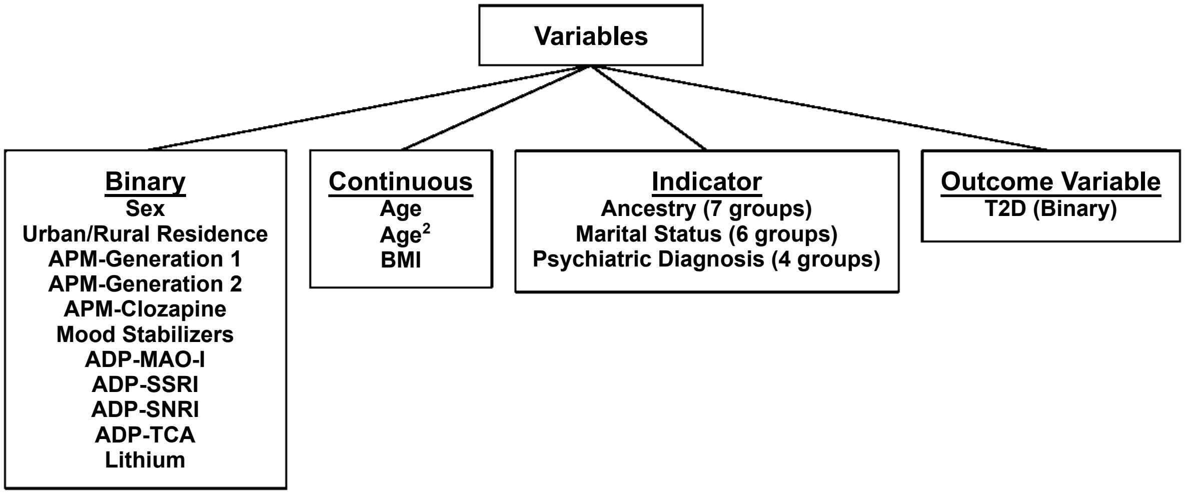

Supplemental Figure 1. Types of Variable Coding.

Age Bin	Sex		BMI				Medication Use			
	Male	Female	BMI Q1	BMI Q2	BMI Q3	BMI Q4	Neither	SSRI	Mood Stabilizer	Both
20s	0.49	0.28	0.07	0.19	0.27	2.27	0.27	0.45	0.29	0.91
30s	2.47	1.87	0.33	0.74	1.99	7.66	1.67	1.96	3.36	3.09
40s	11.41	7.19	2.20	4.50	9.07	23.06	8.21	9.11	12.64	13.62
50s	23.29	16.37	7.38	12.74	22.32	40.66	17.37	19.85	27.93	27.28
60s	32.71	23.13	13.80	23.97	36.03	55.85	25.77	28.68	42.02	39.31
70s	36.50	29.16	17.67	28.62	40.75	59.59	30.17	34.46	50.94	48.74
80s	28.54	25.27	20.60	29.57	39.38	54.37	25.13	30.03	41.14	42.95
90s	19.10	11.23	17.02	24.78	32.58	43.64	16.61	19.96	29.99	32.19

T2D Prevalence

- 0% - 10%
- 11% - 20%
- 21% - 30%
- 31% - 40%
- 41% - 50%
- 51% +

Supplemental Figure 2. Heat plot of Interaction Variables and Prevalence of Type 2 Diabetes Mellitus. BMI quantiles (Q) were determined where bin 1 contain BMI values from 15-24, bin 2 contain BMI values from 25-27, bin 3 contain BMI values from 28-31, and bin 4 contain BMI values from 32-77.
